# Right Pneumonectomy for Right Lower Lobe Lung Cancer with Partial Anomalous Pulmonary Venous Connection in Another Ipsilateral Lobe: A Case Report

**DOI:** 10.70352/scrj.cr.25-0390

**Published:** 2025-09-30

**Authors:** Ryosuke Kaku, Atsuko Watanabe, Makoto Yoden, Mayumi Oshio, Masatsugu Ouchi, Satoru Sawai

**Affiliations:** 1 Division of Thoracic Surgery, Center for Respiratory Diseases, National Hospital Organization Kyoto Medical Center, Kyoto, Kyoto, Japan; 2 Division of General Thoracic Surgery, Department of Surgery, Shiga University of Medical Science, Otsu, Shiga, Japan; 3 Department of Thoracic Surgery, National Hospital Organization Minami Kyoto Hospital, Joyo, Kyoto, Japan

**Keywords:** partial anomalous pulmonary venous connection, PAPVC, right pneumonectomy, lung cancer

## Abstract

**INTRODUCTION:**

Partial anomalous pulmonary venous connections (PAPVC) are rare. We report a case of right pneumonectomy for right lower lobe lung cancer, wherein a PAPVC was detected in the right upper lobe vein before surgery.

**CASE PRESENTATION:**

A 71-year-old man was diagnosed with a mass in the right lower lobe on chest CT. Non-small-cell lung cancer was diagnosed using bronchoscopy. Right bilobectomy (middle and lower lobes) was planned to secure a margin for resection due to hypoplasia of the middle lobe; however, preoperative contrast-enhanced CT revealed a PAPVC involving the right upper lobe. If a PAPVC is identified in a non-resected lung lobe, the patient is at risk of postoperative right heart failure. In cases of PAPVC in a non-resected lobe with Qp/Qs ≥1.5, preoperative or intraoperative revascularization is recommended. Echocardiography demonstrated a pulmonary to systemic flow ratio (Qp/Qs) of 1.39; however, right heart catheterization showed an increase in Qp/Qs to 1.66. Therefore, we considered repairing the right upper pulmonary vein. However, owing to the unstable position of the right upper lobe and the length of the repaired vessel, we performed a right pneumonectomy considering the risk of vascular flexion and occlusion. The postoperative course was uneventful, and echocardiography performed 3 months after the procedure revealed an ejection fraction of 57%.

**CONCLUSIONS:**

In the present case, by assessing Qp/Qs using both echocardiography and right heart catheterization, we determined an appropriate surgical approach. However, right pneumonectomy is a risk factor for right-sided heart failure, and limited resection or nonsurgical treatment should be considered in certain cases. The presence of PAPVC in a non-resected lung requires caution when selecting the surgical approach. In cases of PAPVC, detailed preoperative CT evaluation is essential.

## Abbreviation

PAPVC
: partial anomalous pulmonary venous connections

## INTRODUCTION

Partial anomalous pulmonary venous connection (PAPVC) is a congenital anomaly involving one to three of the four pulmonary veins that abnormally return to the systemic venous system. The incidence of PAPVC is relatively low, ranging from 0.1% to 0.7%.^1,2)^ We report a case requiring right pneumonectomy for right lower lobe lung cancer with the right upper lobe vein draining into the superior vena cava (SVC).

## CASE PRESENTATION

**Fig. 1:**
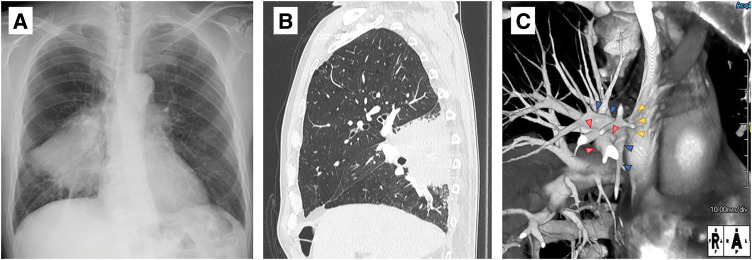
(**A**) Chest radiograph demonstrated a mass in the right hilar region. (**B**) Chest CT revealed an 8-cm mass in the right lower lobe. (**C**) Chest contrast CT revealed PAPVC, with the right upper lobe vein (blue arrow heads) draining into the SVC. The pulmonary artery and the point where the pulmonary vein flows into the SVC are visible (red and yellow arrow heads, respectively). PAPVC, partial anomalous pulmonary venous connection; SVC, superior vena cava

A 71-year-old man was admitted to our hospital after chest radiography during a health checkup revealed a mass in the right hilar region (**Fig. 1A**). Chest CT revealed an 8-cm mass in the right lower lobe and enlarged mediastinal lymph nodes. Fluorodeoxyglucose positron emission tomography (FDG-PET) revealed a mild accumulation in the right lower paratracheal (LN#4R), subcarinal (LN #7), and lower interlobar (LN #11i) lymph nodes. Therefore, a bronchoscopy was performed. Transbronchial lung biopsy (TBLB), endobronchial ultrasound-guided transbronchial needle aspiration (of LN#7), and head contrast MRI were performed. TBLB revealed that the tumor was a squamous cell carcinoma. EBUS-TBNA was performed on LN#7 and no malignant findings were observed. However, direct tumor invasion was suspected in LN#11i and the patient was diagnosed with cT4N1M0, stage IIIA, of right lower lobe squamous cell carcinoma.

Chest CT revealed hypoplasia of the right middle lobe (**Fig. 1B**). Right middle and lower lobectomies were planned to ensure sufficient resection margins. However, contrast-enhanced chest CT revealed PAPVC with the right upper lobe vein draining into the SVC (**Fig. 1C**). We anticipated right heart failure due to an increase in the left-to-right shunt after bilobectomy. Therefore, we opted to perform echocardiography and cardiac catheterization to determine the preoperative pulmonary-systemic blood flow ratio (Qp/Qs). Echocardiography revealed that the ejection fraction was 67%, Qp/Qs was 1.16–1.39, and the wall motion was normal. No heart malformations were observed. However, cardiac catheterization revealed oxygen saturation in the upper SVC of the azygos confluence and right ventricle of 78.8% and 87.6%, respectively, indicating the presence of a right-to-left shunt. Furthermore, cardiac catheterization revealed a high Qp/Q ratio (1.66). We consulted a cardiac surgeon for vein reconstruction but decided not to proceed. This is because the reconstruction would have required a long vein, and it was difficult to predict before surgery when the remaining upper lung lobe would settle with a high risk of obstruction or kinking. At our hospital, we consider preoperative induction therapy in cases where the tumor is large or metastasized to the mediastinal lymph nodes and extensive resection is necessary. In this case, we decided not to administer induction therapy, considering that even if the tumor shrinks, the extent of resection does not decrease; moreover, if the patient develops drug-induced lung injury, radical resection or radiation therapy might become impossible. Therefore, we explained to the patient about his risk of right heart failure and performed a total right pneumonectomy.

### Surgery

**Fig. 2:**
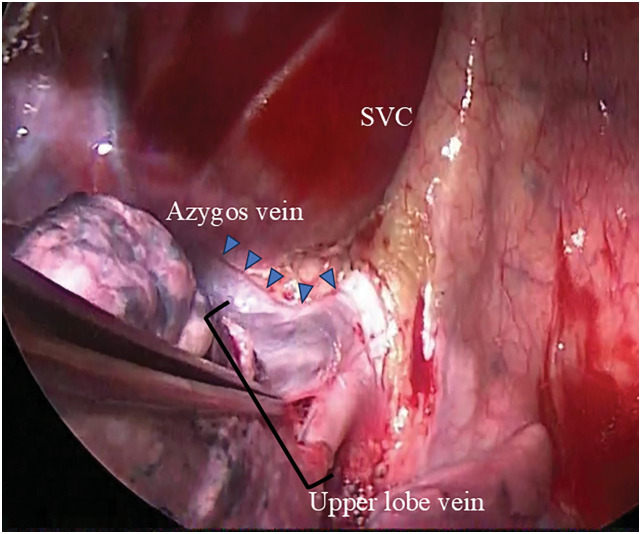
The upper lobe vein enclosed in a black bracket drained into the SVC (blue arrowheads). SVC, superior vena cava

An 11-cm skin incision was made along the anterior axillary line and the thoracic cavity was accessed through the 4th intercostal space. The mediastinal pleura was incised, and the upper lobe vein draining into the SVC was identified (**Fig. 2**); it was subsequently dissected and divided using an automatic stapler (Endo GIA Tri-Staple Technology Camel 45 mm; Medtronic, Minneopolis, MN, USA). The middle lobe vein was ligated and divided, and both the pulmonary artery trunk and inferior pulmonary vein were transected using an automatic stapler (Endo GIA Tri-Staple Camel 45 mm), respectively. The right main bronchus was transected using an automatic stapler (EndoGIA Tri-Staple Black 60 mm), and the right lung was subsequently removed. Additionally, lymph nodes 7 and 10 were dissected. As rapid intraoperative pathological testing confirmed the absence of metastasis, dissection of the upper mediastinum was omitted, and the lymph nodes up to group 2a-1 were dissected. Sealing test before chest closure revealed minor air leakage from the staple line at the bronchial stump. Therefore, an additional U-shaped 4-0 polydioxanone suture was placed to stop the leakage, and the surgery was completed.

### Pathological findings (Fig. 3)

**Fig. 3:**
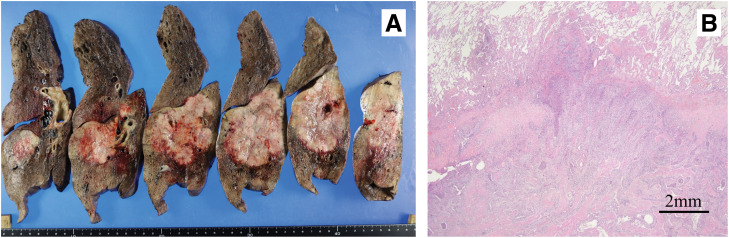
(**A**) The tumor measured 90 mm and was macroscopically observed to have infiltrated into the upper lobe. (**B**) Pathologically, the tumor was diagnosed as squamous cell carcinoma with infiltration from the lower lobe through the interlobar pleura to the upper lobe.

The tumor was identified as a squamous cell carcinoma with infiltration from the lower to the upper lobes across the interlobar pleura. No lymph node metastasis was noted, leading to a diagnosis of pT4N0M0 stage IIIA.

#### Postoperative course

The chest drain was removed the day after surgery. No echocardiography was performed immediately after surgery. Serum levels of N-terminal prohormone level of brain natriuretic peptide (NT-pBNP) was 355 pg/mL before surgery and 458 pg/mL on the first postoperative day, showing only a slight increase. We observed no obvious signs of cardiac failure and the patient was discharged on the POD 7. Given the increased right heart load caused by fluid overload during anticancer drug administration, postoperative adjuvant chemotherapy was withheld and the patient was monitored. One and a half months postoperatively, the patient developed right empyema with a fistula, and echocardiography revealed a small amount of pericardial effusion. However, the ejection function remained at 57%, and the Qp/Qs was 1.19, with no significant increase. Serum NT-pBNP level was 707 pg/mL. Fenestration was performed, and the thoracic cavity became clear; therefore, thoracoplasty was performed. Although no arrhythmias, such as atrial fibrillation, were found, the patient developed cerebral infarction due to carotid artery stenosis after surgery and became bedridden; therefore, cardiac function tests were not performed thereafter. Four months after surgery, recurrence of metastasis to the left lung was observed; however, as the patient was bedridden, anticancer treatment was not possible and he died 2 years after surgery.

## DISCUSSION

PAPVC is a relatively rare congenital malformation in which a portion of the pulmonary vein drains into the systemic circulation instead of the left atrium, resulting in a left-to-right shunt.^1–3)^ The most common site of abnormal venous return was the superior vena cava (SVC; 35.8%), followed by the left brachiocephalic vein (20.6%), right atrium (19.6%), left SVC (7.6%), inferior vena cava (4.3%), azygos vein (3.3%), and other sites (8.8%).^2)^ The left-to-right shunt ratio was 1:10.^4)^ In a retrospective study of 45538 multi-detector row CT cases by Ho et al.,^3)^ the incidence across the lung lobes was 47% in the left upper lobe, 38% in the right upper lobe, 13% in the right lower lobe, and 2% in the left lower lobe.

PAPVC may progress asymptomatically and can be detected during lung cancer resection, as was observed in this case. A Qp/Qs ratio of 1.5–2.0 is considered an indication for surgical intervention.^5–8)^ If a PAPVC is present in the non-resected lung, there is an increased risk of right heart failure due to exacerbation of the left-to-right shunt following resection. Singhal et al.^9)^ proposed treatment guidelines for diagnosing PAPVC before lung cancer surgery. If the PAPVC is in the resected lobe, ligation is performed during surgery. However, if the PAPVC is in the non-resected lung, management is classified into the following three types according to the Qp/Qs ratio: If Qp/Qs = 1.0, the condition is considered normal or asymptomatic and lobectomy or segmentectomy is performed as usual. If 1.0 < Qp/Qs ≤ 1.5, PAPVC reduction surgery may be considered depending on the presence or absence of symptoms and the extent of resection. If Qp/Qs > 1.5, or symptoms are present, revascularization surgery for PAPVC is performed simultaneously with or before lung cancer surgery.

Watanabe et al.^10)^ reported a case of right lower lobectomy for T1cN0M0 cStage IA3 lung cancer with a PAPVC in the right upper lobe. In this case, Qp/Qs was 1.64, which was similar to that in our case; however, as the intraoperative mean pulmonary artery pressure remained stable, the patient underwent right lower lobectomy without revascularization, and no increase in the right heart load was observed during follow-up. By contrast, in our case, concomitant resection of the middle lobe and revascularization would have been necessary if the right upper lobe had been preserved.

Nakata et al.^11)^ reported six cases of lung cancer with PAPVC surgically treated with pulmonary vein reconstruction. Two patients of PAPVC presented with the right upper lobe vein flowing into the SVC; however, in both cases, the tumor was located on the left side, and the clinicians did not have to consider the location or twisting of the remaining lung after surgery, which is different from our case.

In addition, the presence of PAPVC may be overlooked in the absence of a CT scan. Lung resection without recognizing the presence of PAPVC may lead to severe right-to-left shunting, potentially resulting in death.^12)^ In our case, PAPVC was diagnosed using preoperative CT. Therefore, we were able to conduct a thorough preoperative evaluation. Although the patient was asymptomatic, cardiac catheterization revealed a high Qp/Qs of 1.66. However, considering the length of the reconstructed vein, the position of the remaining upper lobe, and the risk of occlusion or tortuosity of the reconstructed vein, we decided to proceed with right pneumonectomy without revascularization. Right pneumonectomy carries the risk of right heart failure and is generally avoided, if possible. Unfortunately, the patient developed empyema with a fistula and cerebral infarction due to carotid artery stenosis, making postoperative anticancer therapy impossible. No signs of arrhythmia or heart failure were observed.

In this case, no cardiac function issues were observed, and the patient’s preoperative performance status was favorable. Consequently, right pneumonectomy was performed to effectively treat the condition. However, depending on the size, location, and pathological type of the tumor, a reduction in the resection area, preoperative induction therapy, and definitive radiochemotherapy may be considered.

## CONCLUSIONS

We report a case of right lower lobe lung cancer complicated by PAPVC in the ipsilateral lobe in which a right-to-left shunt was evaluated using right heart catheterization before surgery, leading to the decision to perform a right pneumonectomy. Although PAPVC is relatively rare, it can lead to serious consequences if overlooked; therefore, a detailed evaluation of the hilar vessels before surgery is considered important.

## DECLARATIONS

### Availability of data and materials

All data generated or analyzed during this study are included in the published article.
